# Hemodynamic management in liver transplantation: toward an evidence-based perioperative strategy

**DOI:** 10.1016/j.bjane.2025.844621

**Published:** 2025-04-01

**Authors:** Andre P. Schmidt, Eduarda S. Martinelli, Virgínia C. de Moura, Liana M.T.A. Azi

**Affiliations:** a Hospital de Clínicas de Porto Alegre (HCPA), Serviço de Anestesia e Medicina Perioperatória, Porto Alegre, RS, Brazil; b Santa Casa de Porto Alegre, Serviço de Anestesia, Porto Alegre, RS, Brazil; c Hospital Nossa Senhora da Conceição, Serviço de Anestesia, Porto Alegre, RS, Brazil; d Universidade Federal do Rio Grande do Sul (UFRGS), Programa de Pós-graduação em Ciências Pneumológicas, Porto Alegre, RS, Brazil; e Universidade Federal do Rio Grande do Sul (UFRGS), Programa de Pós-graduação em Ciências Cirúrgicas, Porto Alegre, RS, Brazil; f Faculdade de Medicina da Universidade de São Paulo (FMUSP), Programa de Pós-graduação em Anestesiologia, Ciências Cirúrgicas e Medicina Perioperatória, São Paulo, SP, Brazil; g University Health Network, Toronto General Hospital, Department of Anesthesia and Pain Management, Toronto, Canada; h University of Toronto, Temerty Faculty of Medicine, Department of Anesthesiology and Pain Medicine, Toronto, Canada; i Universidade Federal da Bahia, Hospital Universitário Professor Edgard Santos, Departamento de Anestesiologia, Salvador, BA, Brazil

Liver transplantation remains one of the most complex surgical procedures in modern medicine. The profound physiological changes experienced during and after orthotopic liver transplantation challenge anesthesiologists and intensivists to provide individualized, evidence-based perioperative care. Among the various aspects of this care, the optimization of fluid balance and hemodynamic stability stands out as a determinant of postoperative outcomes. The Brazilian Journal of Anesthesiology (BJAN) publishes in this issue a set of contributions that deepen our understanding of perioperative hemodynamic management in liver transplant patients, offering timely insights into monitoring strategies, fluid balance, and the prediction of hypotension in this unique population.

In a robust cohort study by Lobo et al.,^1^ the role of postoperative fluid balance is thoroughly examined in 73 adult patients undergoing liver transplantation. Patients were stratified by cumulative fluid balance at 72 hours postoperatively: negative (lowest), moderate (0–2000 mL), and high (> 2000 mL). The findings demonstrate a striking U-shaped association between fluid balance and hospital mortality, with both extremes associated with increased risk. The highest fluid balance group exhibited a 40.5% mortality rate and elevated SOFA scores, particularly linked to primary non-function of the graft and sepsis. Moreover, day-3 fluid balance emerged as a strong independent predictor of all-cause mortality. These findings not only underscore the prognostic value of fluid balance but highlight the importance of early individualized fluid strategies tailored to preserve organ function and mitigate complications in the critical window of postoperative care.

Complementing this investigation, Cywinski et al.^2^ explored the intraoperative use of Hypotension Prediction Index (HPI) software during liver transplantation. Their retrospective analysis of 23 patients undergoing liver transplantation, monitored with both pulmonary artery catheter (PAC) and HPI-enabled arterial waveform analysis, sheds light on the utility of predictive hemodynamic monitoring in this high-risk population. Although the HPI software demonstrated high sensitivity (96%) for hypotension prediction, its specificity and positive predictive value remained limited (33%), resulting in a high burden of false alarms. Furthermore, there was poor agreement between HPI-derived cardiac output (CO) and systemic vascular resistance (SVR) compared to PAC measurements. These findings caution against sole reliance on HPI technology during liver transplants and suggest that traditional tools, such as PAC and transesophageal echocardiography (TEE), remain relevant components of intraoperative management.

This last study is thoughtfully contextualized by a pair of “Letters to the Editor”. In their correspondence, Vetrugno et al.^3^ highlight key physiological considerations, such as cirrhotic cardiomyopathy and abrupt shifts in vascular tone, that complicate hemodynamic monitoring in liver transplants. They emphasize that predictive algorithms like HPI must be interpreted in light of liver transplant-specific pathophysiology and propose that phase-specific performance (e.g., anhepatic vs. reperfusion phases) be evaluated to better characterize the utility of these technologies. Moreover, they argue for a more nuanced approach to interpreting hemodynamic data, warning against generalizations and encouraging tailored monitoring strategies based on surgical phase, MELD score, and clinical context.

In response, Cywinski et al.^4^ reaffirm the exploratory nature of their study and acknowledge the limitations raised. They clarify that their intent was not to validate HPI as a substitute for PAC, but to evaluate its concordance and predictive performance. Importantly, they agree that cirrhotic patients undergoing liver transplantation represent a uniquely complex population, where multifactorial causes of hypotension demand multimodal monitoring. They advocate for further research to define the precise role of HPI software in liver transplant settings and reiterate the need for caution when integrating novel technologies into such delicate clinical scenarios.

Together, these contributions build a compelling narrative: while technological advances in monitoring offer promise, their implementation in liver transplantation must be cautious, contextualized, and ideally, multimodal. Additionally, the importance of individualized postoperative fluid strategies is reinforced, particularly within the first 72 hours, where either excess or deficit can be deleterious. The integration of dynamic monitoring tools, fluid responsiveness assessments, and organ-specific markers may help shape more refined and effective perioperative pathways.

Standard intraoperative hemodynamic monitoring during liver transplantation includes invasive arterial pressure measurement and central venous pressure (CVP) monitoring via internal jugular vein cannulation. Although CVP does not reliably indicate intravascular volume status, directional trends can guide volume management and bleeding risk, especially when assessing portal venous congestion during the dissection phase.^5^ The PAC remains standard in many centers and remains a valuable tool in the perioperative management of liver transplant recipients, particularly those with advanced cirrhosis, complex cardiovascular comorbidities, or challenging intraoperative hemodynamics.^6^^,^^7^ In such high-risk patients, standard noninvasive monitors may be insufficient to fully characterize the underlying circulatory status. The PAC allows direct, real-time measurement of critical parameters such as CO, stroke volume, pulmonary artery pressures, mixed venous oxygen saturation, and systemic/pulmonary vascular resistance, offering a level of detail necessary to guide nuanced, physiology-based management.^6^ Its use is best supported by a comprehensive preoperative cardiovascular evaluation, including recent transthoracic echocardiography to identify patients with cirrhotic cardiomyopathy, pulmonary hypertension, or right ventricular dysfunction, in whom invasive monitoring may change perioperative management. While not routinely indicated, the selective use of PAC in liver transplantation, guided by clinical context and preoperative risk assessment, is aligned with modern perioperative care principles.^7^ Importantly, effective use of PAC-derived data requires anesthesiologists and intensivists to be adequately trained, not only in catheter placement and waveform interpretation, but also in translating findings into therapeutic decisions. When properly applied, PAC monitoring can aid in differentiating types of shock, titrating vasoactive agents, and improving patient outcomes in this uniquely vulnerable population.^7^^,^^8^

Minimally invasive methods for cardiac output monitoring, including transpulmonary thermodilution (PiCCO) and uncalibrated arterial waveform analysis (e.g., FloTrac or Acumen IQ systems), are increasingly used in liver transplantation as alternatives to PAC, particularly in patients without significant cardiovascular comorbidities or advanced cirrhotic cardiomyopathy. These technologies require only central venous and arterial access, offering continuous hemodynamic data with lower procedural risk. However, evidence suggests that their accuracy and trending ability may vary. In a prospective study comparing PAC and the EV1000 transpulmonary system, Vetrugno et al. found acceptable agreement in CO measurements during liver transplants, with a percentage error of 35% and good concordance in polar plot analysis, suggesting that calibrated systems may offer reliable alternatives in lower-risk cases.^9^ Similarly, a large retrospective cohort study comparing PAC and FloTrac-based monitoring reported no significant differences in acute kidney injury, early allograft dysfunction, or 1-year survival, supporting the feasibility of using less invasive devices in appropriately selected patients.^10^ Conversely, Feng et al.^11^ highlighted limitations of PiCCO in trending dynamic parameters like Systemic Vascular Resistance Index (SVRI) and Stroke Volume Index (SVI) during critical intraoperative phases, with high percentage errors exceeding 50% and poor agreement in polar plot analyses, particularly during the neohepatic phase. These findings suggest that while minimally invasive systems may be safely employed in liver transplant recipients with stable cardiovascular profiles, their use demands cautious interpretation, especially during rapid hemodynamic transitions. Ultimately, careful preoperative evaluation, including echocardiography and MELD/CHILD score stratification, should guide the choice of monitoring technique, with PAC reserved for high-risk patients and minimally invasive alternatives used judiciously in standard-risk scenarios.

The use of TEE has significantly expanded in the context of liver transplantation, driven by its ability to provide real-time, detailed assessment of cardiac function, volume status, and acute intraoperative complications. TEE enables prompt identification of cirrhotic cardiomyopathy, right or left ventricular dysfunction, pulmonary embolism, intracardiac thrombosis, left ventricular outflow tract obstruction, and patent foramen ovale, conditions that are often difficult to detect with conventional monitors such as PAC or arterial waveform analysis.^12^ During all three phases of liver transplantation (preanhepatic, anhepatic, and neohepatic), TEE allows dynamic evaluation of preload, contractility, and structural integrity, supporting tailored management of fluid therapy, inotropes, and vasoactive agents.^12^^,^^13^ Moreover, Doppler-based techniques can estimate CO, and simplified protocols with as few as 5 to 9 views have proven effective even for non-expert users, allowing rapid diagnosis of life-threatening events and aiding in real-time clinical decision-making.^13^

Despite these advantages, TEE is not universally employed. A systematic review showed that although more than 90% of liver transplant centers in the U.S. report having access to TEE, only 38 to 56% use it routinely, with others reserving it for high-risk or complex cases.^14^ One key limitation is the need for specific training and certification, as image acquisition and interpretation can be challenging without structured education and experience. The guidelines from the Society for the Advancement of Transplant Anesthesia (SATA) strongly recommend that liver transplant anesthesiologists undergo formal training and certification to ensure the competent use of TEE and reduce operator-related complications.^12^

Another area of concern is safety in patients with esophageal varices, present in up to 70% of liver transplant candidates. While esophageal varices have traditionally been considered a relative contraindication, recent studies and position statements report a low incidence of TEE-related complications (< 0.5%), even in this high-risk group, when procedures are performed by trained operators. Although TEE remains contraindicated in patients with grade III esophageal varices or a recent history of variceal bleeding, its overall risk-benefit profile is generally considered acceptable, especially given the potential for early detection of catastrophic intraoperative events.^15^

In summary, while TEE cannot fully replace invasive monitoring tools like PAC, especially for continuous CO measurement, it provides valuable structural and functional insight that can significantly enhance intraoperative safety and therapeutic precision in selected patients. Nowadays, TEE is considered a complementary modality to PAC rather than a substitute. As evidence and clinical experience continue to grow, TEE is increasingly seen as an essential adjunct in the perioperative management of liver transplantation, particularly in high-acuity scenarios where prompt diagnosis and response can influence graft and patient outcomes.

One of the primary goals of advanced hemodynamic monitoring during liver transplantation is to enable a personalized and physiologically guided approach to fluid therapy. During the anhepatic phase, the goal is to maintain a low-to-normal CVP (up to 10 mmHg) to reduce portal hypertension and intraoperative bleeding.^16^ Balanced crystalloid solutions (e.g., Plasmalyte, Normosol) are preferred over 0.9% saline or lactated Ringer's due to risks of hyperchloremic acidosis and impaired lactate clearance in liver failure patients.^17^ The colloid albumin is administered to replace drained ascites and maintain oncotic pressure, though its overall benefits outside hepatorenal syndrome remain uncertain.^18^ During the anhepatic phase, meticulous fluid titration is critical. Complete caval clamping (versus piggyback techniques) may require higher preload and vasopressor support (e.g., norepinephrine, vasopressin, epinephrine), with judicious fluid administration to avoid right ventricle failure after reperfusion.

Effective hemodynamic and fluid strategies during liver transplantation are tightly linked to postoperative organ function, graft perfusion, and patient survival. Altogether, the findings discussed above reinforce that real-time multimodal monitoring, dynamic interpretation of volume status, and phase-specific hemodynamic targets are indispensable to optimizing outcomes across this high-risk population. Recent evidence highlights that not only systemic perfusion but also regional splanchnic hemodynamics, such as portal venous pressure and portal venous flow, are independently associated with early allograft dysfunction. In a recent systematic review, Brown et al.^19^ demonstrated that postreperfusion portal venous pressure > 15–20 mmHg is strongly correlated with higher rates of early allograft dysfunction and mortality, reinforcing the need for intraoperative portal flow assessment and portal inflow modulation strategies to prevent postoperative complications.

Moreover, intraoperative hemodynamic control during critical surgical phases has a direct impact on postoperative kidney outcomes. In a large retrospective cohort, Bieze et al.^20^ found that intraoperative hypotension, particularly during the anhepatic and neohepatic phases, is independently associated with acute kidney injury, with mean arterial pressure < 60 mmHg sustained for > 20 minutes increasing the risk of postoperative renal dysfunction. These findings underscore the importance of phase-specific blood pressure goals, especially during periods of caval clamping and reperfusion, to protect renal perfusion and optimize patient outcomes.^20^ These insights align with and complement the findings from Lobo et al.^1^ on postoperative fluid balance and Cywinski et al.^2^ on monitoring accuracy, supporting a holistic, evidence-based anesthetic approach to liver transplants.

As anesthesiology continues to evolve with innovations in data-driven care, studies like these are essential to balance enthusiasm with critical appraisal. In liver transplants, where physiology is extreme and outcomes hinge on minute-to-minute decisions, evidence-based protocols must be grounded in both pathophysiological insight and real-world clinical performance. We hope this collection of articles will inspire readers to reflect on current practices and pursue rigorous, patient-centered approaches in liver transplantation.

## Conflicts of interest

The authors declare no conflicts of interest.
